# Problems of knowledge, problems of order: the open science field site

**DOI:** 10.3389/fsoc.2023.1149073

**Published:** 2023-11-16

**Authors:** Liora O’Donnell Goldensher

**Affiliations:** Department of Sociology, Virginia Tech, Blacksburg, VA, United States

**Keywords:** open science, data, ethnography, epistemology, democracy

## Abstract

Ethnographers can and should not just *do* or *not do* open science, but *study* the push to share data, instruments, and other research materials as an important moment of change and contest in contemporary knowledge-making and knowledge politics. Following ethnographers of science and technology who have demonstrated the analytic opportunities afforded by moments of scientific controversy, we should treat the places where these calls are made, debated, and taken up as important field sites for ethnographic inquiry. Whenever and wherever the sharing of data, instruments, and research is discussed, planned, done, measured, judged, or regulated, there are powerful claims, visions, and action concerning what makes for facticity, legitimacy, and credibility in both research and politics. From these sites, I argue, we can observe changes to disciplinary and popular understandings of epistemic virtue, or what makes for reliable, factual, or adequately transparent knowledge production. Attention to these sites can also yield important perspectives on the ways that visions of proper research conduct are imbricated with visions of governance. I argue that turning ethnographic methods to studying the open science movement can enable us to do timely scholarship about shifting understandings of facticity, knowledge, information, and governance.

## Introduction

In October of 2022, I sat in a library workshop designed to train faculty at my university in practices of “Open and Reproducible Research.” I toggled back and forth between my Zoom screen and the Center for Open Science (COS) project workspace I was learning to use. Our instructors emphasized that the aim of the workshop was to teach us how to apply a set of valuable practices to our own research routines. In the first module of the curriculum we’d be following, we’d learn data management principles and practices, and we’d become familiar with some of the repositories in which we might store data. In the second module, we’d learn how to find and create reproducible research protocols and methods. We’d learn about how and why to engage in newer practices associated with open science, like peer review of research protocols. We’d learn to share protocols and check them for transparency according to various guidelines. In the third module, we’d learn about how to organize research collaborations—using the COS tool, around which I was clicking. Lastly, we’d learn why and how to share research materials. The workshop promised better research through these principles and tools: “Both of us,” one of our instructors told us, “just want the best research that can be done in the best way.”

*The best research, done in the best way*. As we began our first module, the idea of an optimal, open, and optimally-open way to do research continued to echo. Data management, our instructors defined, “looks like having well-documented and well-organized data, ideally in public repositories.” Storing our data in a personal Dropbox or Google Drive was, they affirmed, better than storing it locally on a laptop, but “what you really want,” the leader told us, “is an archive, some kind of repository that is maintained by librarians, or archivists, or other people who are interested in long-term preservation.” I began to recognize that I was not just in a workshop about technical questions of data storage and retrieval. Instead, I was in a conversation about what makes for good, even ideal, knowledge-making: how long artifacts should persist, where they should reside, and who should manage and see them. Behind the nuts and bolts of file storage lay a set of rich claims about norms, values, and epistemic virtue (Daston and Galison, 2007).

Like the ethnographic “reckoning” (Murphy et al., 2021) with power and representation that yielded new standards for analyzing positionality and reflexivity, contemporary calls for conventions like data- and code-sharing or advance registration of hypotheses and research designs mark an important moment of change in knowledge production. How should ethnography respond to this push for “open science”? In this article, I argue that we should take these claims about the best way or the right way to do research as objects of analysis. Ethnographers should examine the places where open science is defined, made, evaluated, and practiced as field sites. Here, ethnographers can locate contest and struggle over epistemic virtues in contemporary knowledge politics and governance. Whenever and wherever the sharing of data, instruments, and research is discussed, planned, done, measured, judged, or regulated, there are powerful claims and visions about what makes for facticity, legitimacy, and credibility in both research and politics. We can certainly track these contests at the same time as we ask and answer questions about which practices make ethnographic data transparent in the ways we wish it to be, whether and where data should be stored, and to whom it should be made available (Reyes, 2018). By turning ethnographic methods toward the debates, uses, and regulation of open science, we can and should find potent analytic opportunities to study the relationship between ideals of knowledge-making and visions of governance.

In advancing this argument, I reflect on both my own experiences in my university’s very first workshop^1^ training faculty in practices of open science and on broader policies, standards, infrastructures, and organizations of open science. I use this workshop as an entry point through which to consider the networks of actors surrounding the matter of open science—from scholars to philanthropists, software developers to publishers—where ethnographers and our collaborators in disciplinary locations from human-computer interaction to comparative historical sociology can to ask important research questions about facticity, legitimacy, and epistemic virtue.

Open science, an intellectual movement whose emergence is typically dated to the early 2000s, means different things to different people (see Borgman, 2012). Actors have advanced a wide range of motivations, definitions, accounts of practices adequate to those motivations and definitions, and even positions on what terms to use, such as open science (Nielsen, 2011; National Academies of Sciences, Engineering, and Medicine, Policy and Global Affairs, Board on Research Data and Information, and Committee on Toward an Open Science Enterprise, 2018), open research (Nosek et al., 2015), and open data (Chauvette et al., 2019). Some, especially those who call for science to be opened in order to facilitate validation of existing scientific findings, emphasize the possibility of replicating scientific experiments—and the sharing of research protocols and data that would enable replication (Open Science Collaboration, 2015). Still others emphasize the sharing of publicly funded research with wider publics through open access to publications; others the facilitation of easier and faster collaboration across disciplines, institutions, and between career scientists and non-academics; others new measurements of the impact of a piece of research. Projects from data sharing and open access publishing to citizen science and nonacademic participation in research have gone under the name of open science (Fecher and Friesike, 2014). The notion that it is ideal that something be intentionally put in a public place or made accessible, however, is at the heart of many of the most commonly-circulating definitions: open science, according to one of its central proponents, is “the idea that scientific knowledge of all kinds should be openly shared as early as is practical in the discovery process” (Neilsen, 2011). Most commonly, the terms “open science” and “open research” are used to invoke the sharing of not only knowledge products, but the raw materials of their creation (see Nosek et al., 2015; Open Science Collaboration, 2015), such as protocols, data, and instruments.

Why should ethnographers study the push for open science? After all, psychology, not ethnography, is the field in which questions originally arose about how and why to make the raw materials of research available (see Maxwell et al., 2015). This article opens by considering this question, noting the ways that ethnographers have found rich sites for exploration in open science projects, data-sharing practices, and scholarly conversations about—and understandings of—transparency and open data. I then suggest a new focus for empirical studies of open science projects and calls for open science. I argue that ethnographers and other scholars of scientific knowledge should study the sites that are defining and making open science because calls for open science are calls to shift practices of inquiry and knowledge construction—and they are calls to shift the norms that guide and are used to assess these practices. Treating these as field sites would allow ethnographers to bring transparency to what has been called the “transparency movement” (see Elman et al., 2019). Turning to justifications for data-sharing from in and beyond the workshop I attended, I show how calls for open science often contain powerful and novel claims about what research practices make for reliable knowledge. Whether or not open science calls suggest that researchers should aim for verifiability or reproducibility, they are, as I show, calls to do different things in the process of making facts and drawing interpretive or analytical conclusions: to plan research in novel ways, to collect and record data differently, and to share and store the materials of research in new arrangements and new institutions. As sociologists of scientific knowledge have long demonstrated, when those who do the day-to-day business of getting knowledge-making done begin to do so differently, important social and cultural work is going on—and ethnographers have the right methodological tools to examine the shifting practices through which that work unfolds. Through a reading of STS literatures demonstrating how studying moments of dispute and change in scientific practice can yield important insights about epistemic virtue, or understandings of how scientists ought to behave, I show that calls for open science are more than just suggestions that researchers to try out something new in the process of making knowledge. They are also calls for scholars, publics, and policy-makers to use novel standards to recognize true, authoritative, trustworthy, or reliable knowledge. And because shifts to epistemic virtue are moments when moralities, understandings of the right relationship between science and state, and visions of social authority are contested and renegotiated, these calls offer important opportunities for us to consider classic questions about the relationship between knowledge and order.

I then turn to these standards and to the infrastructures in which they are embedded. Calls to share data and research materials are linked to important changes to standards and conventions through which institutions discern the difference between spurious knowledge and reliable knowledge that should be circulated, supported, cited, and used to make decisions. I show that because practitioners often frame open science as a kind of democratic good, especially for use in governance, ethnographers can investigate these standards not only in scientific and research communities, but also in policy-making. Open science initiatives matter, in other words, for changes to the ways that authorities within and beyond the scientific or scholarly community recognize facticity and legitimacy. This makes calls for open science—and the policy changes that have come in response—key moments in both scientific and political constructions of truth and reliability in information. Taking metrics and curricula from my own discipline and from the workshop I attended as an example, I offer some initial suggestions about questions researchers might ask as we follow open science into policy and politics. In the article’s conclusion, I suggest that ethnographically studying claims about what makes for good knowledge in science and governance can both catalyze timely research about the politics of facticity and more rigorously inform ethnographers’ own data-sharing practices.

## Defining and justifying: ethnographic studies of open science

What does it look like to study open science ethnographically? Ethnographers and other qualitative and interpretive social scientists have found open science to be a fruitful object of inquiry in a number of ways. Some study open science projects as sites from which to examine classic problems in research areas wherein ethnography is already a commonly-applied tool. In this approach, an open science project like a distributed computing partnership can be a chance to investigate the techniques and technologies used to know and manage working at large scale—by, for instance, by observing the metrics and tools technical specialists use to make estimates (Ribes, 2014). Likewise, a gathering focused on open-source software documentation can afford an opportunity for ethnographic investigation of documentation as a social and organizational practice (Geiger et al., 2018). Open science projects can be cases of more general phenomena, and analysis of open science can take an approach that emphasizes continuity with existing scientific or technical practice.

Other studies turn more directly toward open science, treating matters such as openness, data sharing, or data reuse as objects of analysis. Some take up conceptual problems raised by calls to open science: which elements of what might count as data are “signal,” which are “noise,” and how does variety in the ways different researchers assign those classifications to the same objects matter? How might use of data be analytically distinguished from reuse? What is the relationship between one or more datasets and one or more pieces of published research? Investigators of these problems note that there are rarely single or simple answers to these epistemological and phenomenological questions (Leonelli et al., 2016; Pasquetto et al., 2017; see also Borgman, 2012; Wynholds et al., 2012). These definitional matters translate directly to practice, and when researchers ask scientists who advocate for or understand themselves to be doing open science, they find great disagreement over not only what openness means, but what goals should drive open science and what scientists should do in opening their work (Grubb and Easterbrook, 2011). Scientists’ senses of when and how it is appropriate or important to either share data and research or try to replicate or reproduce a study are shaped by a range of institutional, technical, and policy factors—disciplinary career pressures and cultures of competition, varying computational demand in research and availability of data repositories, and the constraints on sharing introduced by disparate ties to industry (Levin et al., 2016).

In my workshop, disciplinary variation in what it might mean to do open science was front and center. “Please stop us,” the facilitators told participants at the start of the workshop, “if you are like, ‘hey, how does this make sense for social science, or for qualitative [research]?” They had shared a short definition of open science: “open as much,” they glossed, “as is feasible, while being protective and careful.” Answers to the question of what could be feasibly shared, they noted, might be motivated differently in different disciplines—the business school, for example, might embargo information in order to protect intellectual property, while social scientists might aim to protect human subjects from identification. For some researchers, describing one’s methodology in terms detailed enough to enable reproduction might mean specifying amounts of a reagent—and for others, a number of interviews.

When researchers examine the range of enactments that go under the name of openness, they also find more than disciplinary disagreement over what protectiveness, being careful, and feasible opening might mean—and over the question of whether a maximalist, as-much-as-possible approach is even the right one to take. Disagreement over the question of what makes for careful or protective opening is closely linked to the question of how to define data and its situatedness. Designers of the Platform for Experimental Collaborative Ethnography, for instance, have suggested that digital tools developed for the natural sciences must be redesigned in order to represent the embeddedness of ethical matters in a given ethnographic data object (Fortun et al., 2016, 16). Those who study open data across political economic contexts, meanwhile, find that definitions focused narrowly access to data resources fail to account for the unequal conditions under which those data might be taken up or re-used (Bezuidenhout et al., 2017).

If narrow definitions of data, data use, and data circulation can fail to deeply account for the imbrication of data with people, communities and their “ethical and political imaginaries,” unequal distributions of resources, and histories of exploitation and extraction, then maximalist approaches and mandates to share data can “retrench historically exploitative relations of knowledge production,” too (Okune et al., 2022, 2–3). Disagreement over what it might mean to be careful or protective in opening data is also linked to the difference, as the coordinators of the Open and Collaborative Science in Development Network put it, between approaches that “purposefully acknowledge and seek to redress power relations within a given context” (Okune et al., 2018, 14) and those that do not. The OCSDNet team, for instance, review cases from an international disaster management collaboration between academics, technology developers, and disaster response officials in the Caribbean; an online biological collections-sharing system in Brazil; and a South African Indigenous community’s discussions with NGO and university-based climate change researchers. They find that when projects treat data as fundamentally situated and define “being careful” as aiming to disrupt exploitative arrangements, open science often does not look like putting something into a public place. Instead, it might look like designing deliberately inclusive infrastructures: a shared vocabulary for collaboration, a data and research framework that accounts for variety among participants in the degree to which openness matters or is desirable, or a contract guiding the terms on which a community might negotiate with future researchers who hope to “open up” their local knowledges (Okune et al., 2018, 8).

Not all calls to share about data, meanwhile, call themselves open science. Ethnographers and other researchers whose methods involve sharing social space with research participants have consistently demonstrated how race, gender, and sexuality matter in the process of making not only knowledge, but data. Researchers’ identities and appearances “structure,” as Tey Meadow puts it, all aspects of the “significant interpersonal relationship[s]” that occur in field research (Meadow, 2013, 467). They are “tools” or “resources” that researchers mobilize, sometimes by “amplifying” and sometimes by “minimizing,” sometimes “strategically and actively” and sometimes through “management of others’ reactions” (Meadow, 2013, 476; Schultz, 2019, 184). The conversations enabled and precluded by researchers’ mobilizations of these identities and appearances leave both presences and absences in the resulting datasets. Traces of those relationships and those identities are also embedded in individual pieces of data, as Ann Morning shows in her descriptions of how interview participants cited their perceptions of her race as evidence in the arguments they made in response to her questions (Morning, 2011, 108). Datasets are also shaped by violence that occurs during their making, and as research about sexual harassment and assault in fieldwork has demonstrated, that violence is often omitted by convention from narratives of data gathering (Hanson and Richards, 2017). Different kinds of openness from data-sharing—what Hanson and Richards call “open discussion” of sexual violence (Hanson and Richards, 2017, 601; Su and Phi Hong, 2023), or what Victoria Reyes describes as “being open about the who, what, where, when, and why of data collection” (Reyes, 2019, 187)—are often invoked by ethnographers who call for accounts of the social shaping of field research.

These definitions of openness are not the ones institutionalized in policies such as the Biden administration’s 2022 directive requiring open access publication and data-sharing in federally-funded research, or analogous European Plan S directives for research funded by European public grants. Likewise, these definitions—and these practices—were not the ones we learned about in the workshop I attended. Even across the range of disciplinary contexts we discussed, some key inclusions and exclusions persisted: the audience for open science was made up of professional researchers, not groups of collaborators including both academics and non-academics. The workflows for opening our research that we reviewed—from registering hypotheses and research protocols in advance to archiving data—all involved putting some research material into a public place, rather than depicting data provenance and associated ethical matters or designing infrastructures to disrupt dispossession. Of course, training professional researchers in the skills necessary to comply with policy protects their ability to do their work without penalty. Yet these inclusions and exclusions legitimate some definitions and enactments of openness, while obscuring others.

In so doing, however, they offer ethnographers rich opportunities to observe struggles over what openness means, what it should mean, and how those meanings might translate into practice. Expanding on maps of definitions of open science that are in use, ethnographers might also trace the paths of divergent definitions and track conflicts between definitions and their disparate implications. When, where, by whom, and with what consequences are disparate definitions of open science, data, use, and reuse deployed? When and where are definitions that are and are not attuned to dynamics of power and exploitation taken up, or replaced with others? Which definitions appear in didactic or regulatory settings, and which are absent or omitted? What happens in moments when multiple definitions collide?

As open science policy continues to proliferate, opportunities for policy ethnography do as well. Analysts have suggested that empirical findings about scientists’ understandings of open science can shape a policy landscape whose guidelines and mandates are new and shifting (Levin et al., 2016, 139), and that findings about what happens *after* data are shared can answer key questions about whether mandates are actually leading to the reuse they hope to foster (Pasquetto et al., 2017). Empirical study of what researchers and nonacademics do with guidelines and mandates can not only yield findings that help refine policy about open science, but can also help us understand how knowledge-making is happening in a moment of major change to the backdrop of rules against which researchers’ day-to-day work unfolds. Knowing that rules and guidelines have unintended consequences, we can also ask another set of ethnographic questions about open science. How do researchers actually navigate the spaces and contradictions between enactments of openness legitimated in policy and others? What do researchers and their collaborators outside of universities do, in the process of their work, with the mandates and guidelines they receive? Do they wholeheartedly adopt them, mimic them even in their absence, flout them, strategically define their data in conversation with them, ignore them, challenge them? If a study of a distributed computing or open-source software project can analyze open science as a case of a broader social and cultural matter like collaboration and documentation, asking questions like these can treat the open science field as a site for the examination of an urgent, and much broader, social and cultural shift: changes to—and struggles over—what makes for good knowledge.

## Suspicion and facticity in calls for open science

Beyond open science’s legitimation by policy, legitimacy matters in another way: calls for open science are often closely linked to calls to recognize some knowledges as legitimate or credible, and others as illegitimate. About a quarter of the way through the workshop I attended, we had learned some technical skills that might be sorely needed for the more disorganized among our ethnographic colleagues—myself surely included—regardless of whether we plan to share data, codes, or research protocols. We had linked our workspaces to an external drive, learned to name our files according to legible conventions and clear directory structures, and reviewed dictionary structures through which to define our data. As a new section of the workshop on reproducible methods^2^ and registrations began, our instructors defined some terms. Reproducible methods and registrations, they glossed, meant “ways to make it so other people can do the same thing as you.” Our instructors suggested we let go of the idea of “getting the same results” as central to reproducible methods. What is most important, they told us, is giving other researchers enough information to enable them to do the same thing you have done, or to understand how you arrived at your result.

Why share what you have done? Our instructors offered a few justifications: they pointed out that thinking through what someone else would need in order to understand one’s research process also produced robust reminders to one’s future self. They flagged equity problems, pointing out that if methodological descriptions are contained only in paywalled research articles, scholars without access to the journals in question cannot learn from the innovations of their peers. They emphasized the possible career benefits of sharing one’s protocols: one’s work is more likely to be read, cited, and included in future research if one’s protocol has been seen. They noted the gaps in the availability of what future researchers would need to build upon a study’s findings that many articles’ methods sections include: in a randomized controlled trial, for instance, it will be difficult for future researchers to follow-up on a comparison of an intervention to “standard methods” if they find themselves wondering just what those standard methods were. And they reported a statistic often cited in open science discussions: that somewhere between 40 and 90% of studies are not replicable due to incomplete reporting of methods.

This figure is at the heart of one of the most common claims uniting often-disparate actors in the open science movement: that “science in practice is problematic” (Breznau, 2021). The problem in question is often identified in terms that equate, or at least associate, ethicality and dependability with the ability to review the raw materials of research and produce the same results as a published paper. For commentators identifying a “crisis of reproducibility,” as Sabina Leonelli writes, “failure to reproduce results…indicate[s], at best, problems in the design and methods used by the researchers in question, and at worst, a fundamental lack of credibility for the knowledge thereby obtained” (Leonelli, 2018, 2). One sociologist, for instance, described a “crisis” of science that is “less reliable, reproducible, and ethical than policymakers, the public, and other scientists expected or previously believed” (Breznau, 2021, 2). Calls for open science, including some calls in my own discipline of sociology, often show their close associations with the related phenomena of “replication crisis” (Maxwell et al., 2015; Shrout and Rodgers, 2018) and “credibility revolution” (Vazire, 2018) by opening with accounts of scandal in psychology, economics or political science. Their citations of exposures of fabricated results (Broockman et al., 2014) and falsified and selectively reported data (Herndon et al., 2014; Kotlikoff, 2018) raise a common specter (see Breznau, 2021): *what if researchers cannot be presumed to be acting in good faith?* By citing disciplinary conversations in which analysis of the hazards of common research practices like “p-hacking” (Simmons et al., 2016) was closely followed by revelations that large swaths of a discipline’s published literature contain spurious, impossible-to-replicate findings, they raise another (see Engzell and Rohrer, 2021): *what if none of this is true*?

Ethnographers might well recognize these concerns. Despite disavowals (see Tsai et al., 2016; Freese and Peterson 2017, 159; Murphy et al., 2021, 43) of replicability or reproducibility as a goal of open research practices in ethnography, calls for open science are haunted by this sense that in order to know that facticity has been achieved and that malfeasance is not occurring—that researchers are not lying in their reports of facts, analytical conclusions, or interpretations—we need more than trust. In the specific world of ethnography, as Murphy, Jerolmack and Smith emphasize, calls for data-sharing have also often been justified with reference to a sense that ethnographers may not reveal enough about our methods and motivations, and that our disciplinary conventions of anonymity and fieldnote privacy “arouse suspicion that the researcher may have something to hide” (Murphy et al., 2021, 42)—and that something may be misconduct or fabrication (see Singal, 2015). Without being shown where researchers went, who they talked to, and why, this line of reasoning suggests, we cannot really know that what they claim is true or that they are trustworthy actors. Echoing concerns that science faces a “crisis of replicability,” the sense that ethnographers might not be adequately achieving facticity haunts many calls for open ethnographic data.

Replication and reproduction have been critiqued as either universal guiding goals of opening science or universal measures of reliability in research—indeed, reproducibility itself has different meanings in different contexts (Leonelli, 2018, 3–4). And many calls for open science suggest that more revelation of scientists’ tools and raw materials will improve research in ways that have little to do with replicating, reproducing, or demonstrating the steps of research in service of credibility. In addition to the equity, discoverability, and research development benefits my workshop highlighted, others argue that sharing data and research procedures will just make for faster, more accurate, more creative, and more advanced science. Using an analytic tool another team has already road-tested, the reasoning goes, simply increases research efficiency, lowers the likelihood of errors, and allows for more advanced methodological development when compared to starting from scratch. Our workshop leaders, shared an example of protocol sharing across disciplines, demonstrating the ways research protocol sharing enabled researchers extracting RNA from primary cortical neuron cultures to adopt a method originally developed by researchers of fish parasites. In disciplines outside the natural sciences, scholars in STS and anthropology have suggested data sharing for the possibility it offers of revealing unforeseen insights by encouraging open-ended collaborations across time and space and multiple interpretations of the same data. The collaborations enabled by open methods and digital archiving of data, they suggest, might traverse disciplinary and methodological boundaries, and carefully-constructed platforms might make it possible to credit a broader range of interlocutors and non-academic collaborators for citable data contributions (Fortun et al., 2016, 3; Okune et al., 2022, 6).

But other dominant framings of the problem that open science seeks to solve invoke, as Moody, Keister, and Ramos put it, Louis Brandeis’ assertion that, “sunlight is the best disinfectant” (Moody et al., 2022, 74). Restricted access to scientific artifacts is sometimes taken as a kind of smoking gun, “interpreted,” as Alicia Grubb and Steve Easterbrook write, “as an indication that scientists have something to hide, thus decreasing their credibility” (Grubb and Easterbrook, 2011, 1). Maybe scientists are acting badly, but under cover of darkness. Maybe their bad action is unfolding in ways we—their colleagues, the public, the funders and taxpayers who give them money, the policymakers and healthcare providers and regulators who use their findings to inform practice and lawmaking—cannot perceive. Maybe, by that concealed bad action, scientists have been finding not facts, but untruths. And maybe, then, our follow-up research, our sense of how the world works, our policies, our healthcare interventions, and our regulations are built not on the solid foundation of truth, but on the shifting sands of fabrication, misuse of methodological tools, *post-hoc* hypothesizing, and opportunistic decisions to exclude data and selectively analyze or report variables, measures, and research conditions. This possibility preoccupies many calls for open science, and it drives the sense that there is a relationship between facticity, legitimacy, and the degree to which others can see what scientists did to arrive at their results.

Inadequate revelation of what scientists did to arrive at their results is just one way this problem is identified, and our instructors encouraged us to loosely hold the goal of reproducing the same results. But as they suggested particular tools for registration, the sense lingered that part of what these tools could offer was, to be colloquial, receipts: “By preregistering a protocol and having a timestamp and a [record of] versions,” one told us, “you have evidence that ‘hey, I planned this analysis ahead of time. I did what I said I did.” This, they told us, could help avoid accidental p-hacking—but it could be useful for qualitative research, too. If researchers register plans and data collection, for instance, they told us, then “we know that this was for real.”

*This is for real*, and *I did what I said I did:* implicit in specific calls for data-sharing in ethnography and many general calls for open science is a sense that there is a set of practices that, if used correctly, could reliably yield facticity and legitimacy. To believe that truth has been found, calls for open science often suggest, we need to see that something particular has been done. But ethnographers need not only respond to the suspicion of calls for open science or for data-sharing in ethnography by amending our own practices. Instead, we can treat this suspicion—and the suggestion that often accompanies it that there are correct ways to act in order to find facts—as a signal that open science is a site where we can study the potent relationship between moralities and facticity.

## Doing science the right way: epistemic virtue

For the past half-century, sociologists of knowledge and scholars in and near the field of science and technology studies (STS) have challenged the idea that facts are out there to be discovered, if only we do things right, and that researchers are extracultural figures who could avoid corrupting science with social practice. These scholars have suggested that if we want to understand where facts come from, we can attend to what scientists *do*, the cultural settings in which they do it, and the normative precepts by which they discern what *doing things right* might mean. This might involve, in the tradition of laboratory ethnography, observing the ways that individual scientific facts are distinguished from alternatives—not purely deductively or logically, but instead by attaining meaning and significance in “microprocesses of negotiation” between human investigators and nonhuman phenomena from laboratory layouts to schedules (Latour and Woolgar, 1979, 42, 135, 145–6, 158). In letting go of the idea that there is a realm of pure science outside of culture where truth is discovered, ethnographers gain the chance to observe the ways that a scientific discipline is a distinctive cultural setting with its own conventions and patterns, “cultural machineries,” ontologies and semiological systems, and understanding of “what it means…to work empirically” or to measure (Knorr-Cetina, 1999, 9–12).

Many powerful studies of scientific action have attended to the unfolding of the everyday process of fact-construction, but moments of disruption afford particular opportunities for analysis. Such moments include both emergence—“paradigm shifts” and moments in which new forms of scientific thought evolve (Fleck, 1979; Kuhn, 2012)—as well as conflicts, disputes or controversies (Latour, 1987, 258; Nelkin, 1992). Scientific disagreements are good places to understand the social and cultural work of settling or stabilizing facts. But in controversies over the formation of new fields like genetics or climate science, we have natural laboratories for observing whole belief systems, methods of inquiry, or perceptions of reality (see Fleck, 1979; Edwards, 2010; Hilgartner, 2017).

This latter kind of dispute is a normative, even an ethical one: it is over the unsettled question of what scientists will recognize as “epistemic virtue” (Daston and Galison, 2007, 16). Controversy gives us the chance to ask a key question: “How and why were certain practices and beliefs accounted proper and true?” (Shapin and Schaffer, 1985, 14). Practices and ideals that we might now take for granted as the right ways to act or the right ways to observe in pursuit of scientific fact—using experiments to produce credible proof, for instance, or aiming for objectivity in representations—are, these scholars have shown, in fact the *ends* of dispute (Shapin and Schaffer, 1985, Daston and Galison, 2007, 17–18). They are forms of conduct for which scientists began to strive in a particular moment, and they are definitions of the way scientists ought to behave that have often coexisted with others. Controversy studies have shown that where there are claims that a particular sort of conduct in science is right and another kind is suspect or wrong, there is analytic opportunity.

Some time after the workshop I attended, I sat down to review a journal article and found myself remembering something. One of the ways to we’d learned to identify the “baseline” of what needs sharing by way of data or analytic tools was to think about what would be necessary in order to review a paper. “It’s often difficult [when] there’s not enough data and code to see what actually happened and tell whether they are doing a good job,” our instructor elaborated, leaving reviewers to “sort of tease it back out from the tables.” Remembering this, I found myself thinking about the different ways we might identify the right way to do peer review. When I review an article—a task I am more likely to do for a qualitative than a quantitative paper, thus leaving me with fewer tables to tease out—do I see it as part of the purview of my task to not just evaluate a description of the methods used, but to follow and evaluate each step of analysis? It struck me that behind this definition of the task of reviewing, too, was a particular moment in which epistemic virtue was defined, a particular definition of what it means to see enough to evaluate the quality of research.

It might be tempting to describe this kind of inquiry as a sort of dodge, or a way to sidestep the hard work of empiricism. Why try to make a “reasonably reliable rendering of the social world” (Duneier, 2011, 2; see Murphy et al., 2021, 43), a skeptic might argue, when we can instead sit back, relax, and declare that there is no such thing? My suggestion that ethnographers study the social and cultural action by which facts are constructed and epistemic virtues are worked out in open science, is, however, not an abandonment of empirical work. Instead, it is a call for more of it. It is not a turn against the idea that something really is happening in the social world, something from which we can learn if we pay attention. Instead, it is a call to understand the sites where knowledge is made and the conditions of its production are debated as part of the social world, deserving of ethnographic attention, and capable of revealing a great deal. Readers might indeed have “a right to a reasonably reliable rendering” (Duneier, 2011, 2) of the social worlds of science and technology—and of the social worlds where prescriptions for the right way to do research are formed.

In the moments when new epistemic virtues arise, and in struggles over the degree to which they will win out, what is hashed out exceeds visions of the right way to act in a laboratory or understandings of the kinds of actions that make for credible knowledge or adequate proof. Instead, as Shapin and Schaffer famously put it, “solutions to the problem of knowledge are always solutions to the problem of social order”: moments in which we see shifts to accepted modes of scientific action are also moments of political contest. Working out “the genuineness of knowledge” takes working out the structure of a community of knowers, and in renegotiations of scientists’ roles and activities, we also see renegotiations of understandings of moral citizenship, of definitions of the relationship of scientists to states and polities, and of visions of the right ways to organize social authority (Shapin and Schaffer, 1985, 332–341). We think of epistemic virtues as if they are self-evident at our own peril. When we treat epistemic virtues as if they have been there all along, we not only misunderstand them, but we also lose analytic opportunities to ask about their emergence and to see what is at stake in the struggles that surround them.

If ethnographers recognize the emergence of open science as a moment of contest over epistemic virtue, we can track the consequential renegotiations of facticity, of social authority, and of morality that such moments inevitably contain. Open science advocates who see a crisis of legitimacy in scientific research have suggested that inadequate facticity can be traced to “researcher degrees of freedom,” or the myriad decisions researchers make in the process of analyzing and even collecting data (see Simmons et al., 2016). Ethnographers and historians of science, however, have turned this proposition on its head, showing how the social action of science does not impinge on or threaten objectivity. If the process by which facts are produced is instead always saturated with the contingency of culture, convention, dispute, and negotiation, then by “playing stranger,” by finding gaps in the taken-for-grantedness and self-evidence of propositions about how to do science, we can gain great analytic opportunity. How might ethnographers take up this opportunity? Ethnographers of open science in practice might observe processes of opening and closing data, analytic tools, and research plans. As they make both programmatic and momentary decisions about what to share and how to share it, how do researchers negotiate within and between teams, definitions of facticity and empiricism, and material qualities of data objects, specimens, and technologies of analysis? How do scientists recognize or identify when they have “done a good job” at openness? How do factors such as researchers’ understandings of their own—and one anothers’—roles, experiences of pleasure or enjoyment, or time matter for the process of opening research materials? Studying open science ethnographically in this way can help us analyze the shifts to practical understandings of facticity, good scientific conduct, and the right relationship between knowledge and governance in a contemporary moment of dispute and controversy.

## Standardizing openness

We can learn a great deal about shifting understandings of epistemic virtue by studying the data- and research materials-sharing practices of individual researchers and teams of researchers. But as scholars of open science who study the platforms and policies of open science have argued, we might also fruitfully study the infrastructures, institutions, and classifications that organize, constrain, promote, and limit those practices. As Stephen Hilgartner puts it, opening and closing research—the “work to control which knowledge become available to whom, when, under what terms and conditions, and with what residual encumbrances”—does not “take place on an open field, unconstrained by history, identity and institutions.” Instead, it is shaped by “regimes of closure” (Hilgartner, 2012, 267, 272). Regimes like these link knowledge and governance through “legal or quasi-legal forms,” are embedded in “institutionalized discourses and practices” from publication and intellectual property law to disciplinary epistemic cultures, and deserve ethnographic attention too.

Critical scholars of open science who analyze infrastructures of scientific work have paid particular attention to platforms and systems for recording and sharing data and workflows. These analysts have emphasized that while some knowledge infrastructures are intentionally built to account for power, others take a one-size-fits-all approach that can retrench extractive and exploitative dynamics. Once infrastructures for open science are in place, they point out, actors do not all benefit equally and scientific work does not automatically unfold equitably (Okune et al., 2018, 2). For scholars in library and information science, studies of organizations, and other fields, documentation work like that of recording the process and materials of research is central to the sustenance of social structures, documentation work is a “mode of collective sense-making,” and it is often through documents that people “make their understandings and intentions known to others” (Geiger et al., 2018, 772–3). If the design and use of knowledge infrastructures matters, these scholars have suggested that their ownership does too. Some analysts of open science have raised concerns that open science platforms could easily be dominated by profit-driven infrastructures owned by commercial firms that have been critiqued for their surveillant and extractive strategies (Sadowski, 2019; Dembicki, 2022, 3; see Pooley, 2022).

If we study open science with a particular eye to epistemic virtue, however, we might pay attention to research infrastructures in another fashion: we might study the ways that they “embody,” as Susan Leigh Star and Karen Ruhleder famously put it, standards and classifications (Star and Ruhleder, 1996) for research conduct. Empirical academic researchers, myself included, now do our work in the context of increasingly prevalent mandates and incentives to share our data—mandates and incentives that flow not directly from our employers, but instead through other institutions. The workshop I attended, for instance, was not required in any way. While it taught me a great deal, especially about the nuts and bolts of organizing my research in ways that might enable me to collaborate and retrace my own steps, I attended entirely out of my own interest. Neither data-sharing nor open science training are mandated by my university. And though I earned “professional development” credit for participating, I could have earned the same credit by attending, for instance, a workshop on PDF accessibility, or one about how to use a particular tool in the Canvas online learning site to which my university subscribes. If sharing the raw materials of research is to become a key signal of credibility and legitimacy in research or a key step in our common-sense definitions of doing research the right way, it might not happen because researchers are directly compelled by their employers.

For ethnographers interested in the negotiations of epistemic virtue that unfold around the questions of how and why to share research materials, this means that some of the action we can fruitfully observe may be located around the standards and policies of funding and publication infrastructures. “If you are doing funded research, US government funders almost without fail—and increasingly, other funders—will require these sorts of things,” our instructors told us. Openness standards are increasingly woven into disciplinary work at the level of publication, too. In my home discipline of sociology, major journals I frequently use in my research and teaching have adopted a standard set of Transparency and Openness Promotion guidelines (Nosek et al., 2015), including *Sociological Methods and Research* and, by virtue of an Elsevier-wide policy, journals such as *Poetics, Social Science and Medicine, Social Networks*, and *Social Science Research* (see Breznau, 2021, 8).

In funding and article submission workflows that encode incentives or penalties based on standards like the Transparency and Openness Promotion guidelines, we see infrastructure that embodies “judgements about what constitutes a legitimate intellectual contribution, for whom, and with what implications” (Levin and Leonelli, 2016, 283). Ethnographers of standards and standardization have emphasized that standards do not implement themselves: instead, they are “tinkered with” in practice and rarely work as their designers intend them to (Timmermans and Epstein, 2010, 81; Timmermans and Berg, 2003). If ethnographers of open science treat the trajectories of these judgments standards as indeterminate, we have the chance to ask important questions: How, we might ask, are standards for adequate transparency and platforms for sharing data and other research materials actually being used in scientific spaces like laboratories and research teams? What forms of sense-making and what social structures in research communities are maintained through the use of publication and funding infrastructures that incorporate standards for data-sharing? How and when are these platforms and standards used in novel or unintended ways, navigated strategically or creatively, hacked or expanded? How is their use embedded in and enrolled in coordinating the day-to-day work of research? As they use research infrastructures that incorporate standards for open science, what do researchers actually do with the judgments they embody about what makes for intellectual legitimacy? And what do researchers do when they are caught between multiple standards for sharing research materials, or between standards for data-sharing and other normative systems?

Regimes of closure, Hilgartner reminds us, are multiple (Hilgartner, 2012, 274). Classifications and standards, meanwhile, emerge from action. “Someone, somewhere, must decide and argue over the minutiae of classifying and standardizing,” write Susan Leigh Star and Geoffrey Bowker (Bowker and Star, 2000, 44–5). The indeterminate histories of competing classifications and standards for open science are scenes of negotiation we might fruitfully observe. In disputes and negotiations over standards of openness, ethnographers have a natural laboratory of controversy and change in which to observe constructions of epistemic virtue. By studying when and how classifications and standards of openness are deployed, undermined, challenged, ignored, tinkered with, and embraced, we can treat mandates to share data and standards for openness not as automatic conduits for new definitions of epistemic virtue, but instead as rich sites from which to observe what happens to those definitions in scholarly and scientific practice.

## Data-sharing for governance: open science and the problem of social order

Yet when scientists and other researchers publish findings, their legitimacy is not only appraised by funders, publishers, or other scholars. Classifications of research as credible, trustworthy, or done the right way reverberate beyond the laboratory, seminar room, or journal page: they are also closely related to the question of whether that knowledge will be treated as publicly and politically legitimate, well-enough-made to be used in governance. In studying calls for open science as moments of struggle over epistemic virtue, we can also track these calls—and the standards and infrastructures that surround them—beyond academic settings. In the following example, I offer a number of lines of inquiry that ethnographers might pursue in investigating open science’s significance beyond scholarly settings.

The idea that open science has something to do with politics is a common one. Development of those same Transparency and Openness Promotion guidelines now used by many sociological journals, for instance, was a central and early project of the Center for Open Science (COS), a major open science organization founded with the goal of transforming the process of research and the ways research findings are assessed and used in policy-making. Although far from the only organization whose activities and initiatives sustain practices of sharing data or research materials, COS, which both hosts the Open Science Framework repository site I learned to use in my university’s workshop and created the template for the workshop’s curriculum, has become a major force in the creation of both standards and platforms for open science. The organization was launched in 2013 by philanthropists Laura and John Arnold’s Foundation. The Arnold Foundation remained, with the Department of Defense agency DARPA and charities established by the late investor and philanthropist John Templeton, one of its three largest funders by 2019.^3^ The organization’s work is guided by a fairly radical vision of data-sharing: “direct access” by “default” to the data used by scholars to support their claims and preservation of “all scholarly content” (Center for Open Science, n.d.). Founding COS is part of what Laura Arnold described as the Foundation’s “aggressive investment” in “evidence-based policy” (Arnold, 2017). The Arnolds’ work aims to promote policy-making guided by research findings—not just any research findings, but research findings whose legitimacy is confirmed by their use of particular methods, by the publicity of their research materials, and by their replicability.

In a 2017 Tedx talk, Arnold made the case for open science as an essential element of the solution to what she called a key policy problem: “We’re routinely making all kinds of decisions based on incomplete, inconsistent, flawed, or even nonexistent data,” she said. “These shenanigans happen everywhere.” Arnold summarized the Reproducibility Project that spurred psychology’s “replication crisis,” another project of COS led by its co-founder and director Brian Nosek (Open Science Collaboration, 2015). “We asked researchers to reproduce 100 psychology experiments that had been published in top psychology journals in 2008,” Arnold explained. “You know how often they could find the same results? One third to one half of the time.” This publication of unsuccessful attempts to replicate psychological experiments, Arnold said, demonstrated “something that we see throughout academic research and throughout research in general: virtually everywhere you look, you’ll find researchers, many of them prominent and most of them well-intentioned, actively misleading us into believing that bad research is proven fact.” This might, Arnold told her audience, not be a problem of intentional fraud. When researchers, for instance, “cherrypick” positive findings on secondary outcomes and report those instead of reporting null findings on their original hypotheses, they might be doing so because of institutional pressures. “The incentive system in science and research is broken,” Arnold went on. “Scientists and researchers are motivated by the desire to publish, which brings tenure, funding, and fame.”

In creating standards like the Transparency and Openness guidelines and securing their adoption by scholarly organizations like major journals, COS aims to not just convince researchers that their instruments, platforms, and metrics are good ones. Instead, by using the power of incentivization to create rewards for conducting scholarly work in ways that align with the organization’s definition of “openness, integrity, and reproducibility of research” and penalties for conducting research in ways that do not, the organization’s strategy seeks to build a scholarly publishing infrastructure that “embodies,” to use Star and Ruhleder’s term, COS’s vision (Center for Open Science, n.d.). But these advocates describe their work as aiming beyond the academy. “We became philanthropists to change the world,” Laura Arnold’s TedX talk went on. If philanthropists can and should change the world, Arnold suggests, they can do so in part by changing the relationship of governance to knowledge. The Arnolds are important advocates for using randomized controlled trials (RCTs) to direct public policy, and they advocate against funding programs that are not supported by what Laura Arnold called this “gold standard” of evidence. “We’re spending millions and millions of dollars on programs that at worst do not work, and at best, we do not know, or we do not have sufficient data or we do not have reliable data. This needs to change, we need to stop.” The foundation’s investments in the Center for Open Science and its work to build standards and platforms for data-sharing, Laura Arnold said, were her Foundation’s attempt to not only shift what she called the broken incentive structure of science, but also to make “healthy data systems” that could be used in making evidence for policy-makers to “follow.” “We try to break this cycle and reform the system,” Arnold continued, “by funding organizations that are promoting transparency and good practices and collaboration and data-sharing.”

What does open science have to do with the ways that knowledge is classified or recognized as good enough to govern with? By tracing the public trajectories of open science projects like transparency standards that aim to classify some research as reliable or credible enough to be used to be used in policy-making and other research as illegitimate or spurious, ethnographers can observe important contemporary contests over epistemic legitimacy in politics—and ask key questions about how the politics of philanthropy shape public uses of science and research. For instance, ethnographers—alongside other researchers, such as political scientists and comparative-historical sociologists—might analyze the success of efforts like the Arnolds’ to define some knowledge as trustworthy enough to be used in policy-making on the basis of particular data-sharing standards. Is a crisis of epistemic legitimacy actually arising from these calls, and if so, what are its terms? How do politicians, regulators, or social movements actually relate to evidence-based policy that cites openness of research as a signal of trustworthiness and reliability? When, where, and how is the mantle of evidence-based policy based in open data taken up? When, and why, are these standards taken up or not taken up in the process of making law or funding public programs? In the day-to-day work of legislatures, regulatory bodies, city council and school board meetings, when is data-sharing is invoked as a sign of credible research—and when is data unavailability invoked as a sign of doubtful legitimacy? Have philanthropic efforts to formalize open science standards and promote open science actually produced a situation in which science or research that does not share or selectively shares data is treated by policy-makers as an unreliable basis for governance? How, exactly, has the flow of foundation funding to standards for research conduct shaped what is actually treated by legislators or regulators as legitimate or illegitimate knowledge, or as good or bad evidence of effective policy-making?

Echoing studies of the evidence-based policy-making to which calls for data-sharing are related, ethnographers might also ask what *kinds* of policy tend to result from using standards for data-sharing to distinguish reliable from unreliable knowledge. A central criticism of policy-making guided by RCTs, for instance, is that it is a technocratic strategy of focusing on small questions, problems, and solutions, and that it tends to lead to less transformative outcomes. Arnold Ventures, the limited-liability corporation the Arnolds created in 2019 to more efficiently accomplish their political goals by combining their Foundation, their donor-advised fund, and their political advocacy group Action Now (see Schultz, 2019), has often explicitly framed their interventions in this way: as intended to avoid more transformative reforms by tinkering with existing structures (Jeffries and Ridgely, 2020). For instance, in the wake of public criticism of the US’s system of policing and incarceration, Arnold Ventures similarly described the research it funds as serving to identify inefficiencies and harms that could be reformed while retaining the existing criminal legal system, which the organization described as offering “real public safety benefits” (Fontaine, 2022). If policy-making guided by RCTs tends to result in “formal, not transformative reforms” (Gilmore and Gilmore, 2022, 316), are there patterns in what tends to be decided or enacted when data-sharing standards are used to validate knowledge for governance?

Ethnographers of open science who follow standards for adequate public sharing of research materials into policy and politics might also compare the negotiations that surround these standards to those that surround other related instruments. For instance, in addition to spearheading creation of the Transparency and Openness guidelines, John Arnold has been a key supporter of pretrial reform campaigns that rely on algorithmic risk assessment tools (RATs) and Arnold Ventures is a key RAT creator. Their Public Safety Assessment, offered free of charge, is used statewide in four jurisdictions and multiple municipalities. These tools have been criticized by a host of community groups, from the NAACP and ACLU to Mijente and National Bail Out, and also in a 2019 consensus statement by 27 researchers at MIT, Harvard, Princeton, NYU, the University of California-Berkeley, and Columbia, who characterized them as racially biased by nature (Whitlock and Heitzeg, 2021, 113–118). Technologies and tools like these do not necessarily ensure equity in decision-making, often instead encoding—and then reproducing in the information they produce—both racial biases and naturalized definitions of racial difference (see Braun, 2014; Noble, 2018; Moran-Thomas, 2020; Liao and Carbonell, 2023). RAT campaigns, meanwhile, compete with others such as money bail reform mandating pretrial release that are supported by broad-based community groups. There is rich action to observe when advocates create standards, metrics, and instruments, then encourage consequential institutions to make them a part of the infrastructures through which they function. Ethnographers of open science might compare data-sharing standards to other objects like RATs, asking what is naturalized in their use, and following whether, how, and why their paths are marked by similar conflict and contest—and whether and why data-sharing standards are more seamlessly accepted.

Lastly, ethnographers and other scholars of open science have another chance to investigate how, to paraphrase Shapin and Shaffer, arrangements of knowledge in open science are related to arrangements of power: we can respond to and expand upon existing scholarship about the relationship of open science to democracy. Scholars of open science often note that it is frequently characterized as a democratic good (Okune et al., 2022, 2). Proponents in what Benedikt Fecher and Sascha Friesike call the “democratic school” hope that open science might democratize research by making its products—both data and publications—more widely available through open data and open access publishing (Fecher and Friesike, 2014, 27–32). This might address problems of equity, as the instructors of my Center for Open Science-developed workshop suggested: even without access to the deep pockets of elite institutions in the Global North, scientists and scholars could learn from the methodological innovations of published papers in their fields that might otherwise be paywalled, and researchers could analyze data—including ethnographic data—without incurring the costs of its collection. Others hope this might happen through “citizen science,” whereby laypeople and noncredentialled members of the public might participate in the research process, and suggest that this participation might yield “radical change to the structures of political power” (Cavalier and Kennedy, 2016, 117; see Mirowski, 2018). Those in what Fecher and Friesike call the “public” school of open science see the openness of these sorts of citizen science projects—along with the horizontality of science communication projects—as dismantling scientific elitism, serving as a “form of devotion” to a broader audience for research.

In practice, open science projects relate to matters of democracy in research—distributions of decision-making power and resources in the scene of data-collection, data-sharing, and data-use—in ways that are more varied and more contradictory than those imagined by either the “democratic” or “public” schools. We have seen how differently decision-making is assigned in open science initiatives in which regulators mandate data sharing and in open science projects that build shared vocabularies or processes for data negotiations between communities and researchers (Okune et al., 2018, 8). Meanwhile, critics have noted that many citizen science projects simply reproduce hierarchical and even exploitative arrangements of knowledge production, with credentialled, career scientists making analytic decisions while laypeople are delegated often unpaid, intermittent tasks (Powell and Colin, 2009; Fecher and Friesike, 2014, 23), and have suggested that if this is a model of democracy, it is a thin one indeed (Mirowski, 2018, 177). The assertion that open science is a democratic good might, of course, mean a number of different things, depending on whether we understand the word democratic to denote, on the one hand, that something relates or is available to a broad mass of people, or on the other, that it is characterized by decision-making or governance by a broad mass of ordinary people (Merriam-Webster, 2023; Brittanica, 2023). Critiques that highlight the question of who makes decisions in citizen or open science projects argue that these research arrangements can privilege the first of those definitions over the second: they might make data, research materials, or the activity of data-collection *available* to a broader mass of people, but they do not always turn the *decision-making* about research over to laypeople. These critics suggest that if we want science to become more democratic, we ought to mean something more significant by democracy than mere access to research materials or participation in any stage of the research process.

If we keep in mind the ways that sharing research materials figures in visions of evidence-based policy-making, however, we can recognize further questions about the relationship between open science and democracy. We can ask about how classifying some knowledge as legitimate and other knowledge as illegitimate on the basis of data-sharing standards is related to the question of who does public decision-making, and to the question of whose voices, accounts, and priorities can shape that process. One approach to this question might begin with the relationship between open science funding and broader philanthropic program in which it arises. While both major private funders of the Center for Open Science are deeply invested in a wide variety of other issues—from the Arnolds’ grants supporting abortion access (Arnold Ventures; Abortion Care Network, 2023) and emergency Head Start funding during the Republican-led 2013 federal government shutdown (CNN, 2013), to Templeton’s foundations’ investments in positive psychology and neuroscience (Templeton Foundation, n.d.; Positive Neuroscience Archives, 2023)—Arnold Ventures and Templetons’ foundations’ investments share another area of overlap. Both have invested deeply in projects steering policy-making away from directions suggested by grassroots organizing, community decision-making, or collective action. Templeton’s foundations, which are one of the chief funders of think tanks and advocacy and trade organizations promoting climate change denial (Brulle, 2014), made one of their largest grants to the Atlas Economic Research Foundation. AERF, a self-described “worldwide freedom movement” also funded by the Koch brothers and oil companies such as ExxonMobil, has been credited with convincing Canadian state officials to limit Indigenous communities’ ability to prevent oil pipelines and fracking operations by refusing to sign on to a United Nations declaration ensuring Indigenous peoples’ right to consent to fossil fuel extraction on their lands (Dembicki, 2022). Arnold Ventures’ political work, meanwhile, has also taken direct and targeted aim at the power of public employee and teachers’ unions, advancing school privatization through charter advocacy (Support Our Public Schools, n.d.; Ballotpedia) and “dominating” the funding of efforts to slash public employee’s pension benefits in favor of private investment in riskier assets like hedge funds (Frying Pan News, 2013). Recalling the second definition of what it might mean for something to be democratic, we can investigate the relationship between visions of good policy-making that use data openness as a marker of legitimacy and the extent to which governance is done by broad mass of ordinary people. If and when the openness of research or information is indeed invoked as a marker of epistemic legitimacy in policy-making in the ways suggested by the projects these funders support, are there patterns in how these appeals bolster or erode the degree to which governance unfolds democratically, or on the basis of collective action by ordinary people? Do open science standards for good knowledge in policy-making change or limit the voices or perspectives admitted to the process of public decision-making? Is the power of ordinary people to use observational and experiential accounts of their lives and lands to shape what happens in their jobs or their homes increased or decreased by standards for data-sharing in policy and politics?

As sociological analysts of philanthropy have long noted, heterogeneity in funding and incongruity between funded projects and philanthropists’ own political identifications and agendas are both common (see Karl and Katz, 1987). Questions of how to define open science and which research practices are proper are, however, closely related to questions of governance. Who should make decisions not just about what happens in the course of research, but about what happens in the world? What kinds of information and knowledge should guide those decisions? And what is the right way to do the work of making that knowledge? Through their close relationship to standards for legitimate policy-making, standards of legitimacy and facticity “embodied” in key open science infrastructures link the problem of knowledge to the problem of order. These links deserve our ethnographic and critical attention.

## In the laboratory of dispute: toward ethnographies of facticity

In moments of disagreement about scientific inquiry or change to the practices and ideals by which science is done, we have unique access to the ways that actors ask and answer questions about how science *should* be done. As calls for and practices of open science become more widespread and more deeply institutionalized across disciplines, ethnographers, I have suggested, should study open science with an eye to the way that it is a site of debate, struggle and negotiation over epistemic virtues in contemporary knowledge and governance. I have argued that ethnography benefits by following STS and ethnographers of science and knowledge in treating the open science moment of change and dispute regarding both scientific practice and epistemic virtue as a kind of natural laboratory.

Practices of open science like public data storage are often suggested as tactics that can shore up the facticity of research, including ethnographic work. I have argued that ethnographers can treat open science calls and practices not just as chances to prove that we are not lying, but as provocations to treat facticity as an object of our study, and we can treat the sharing of research materials as not only a strategy to adopt, but a knowledge practice to study. Indeed, the profusion of available data, instruments, research designs, code, and more across differing platforms and audiences that has resulted from open science policies and practices offers itself for ethnographic analysis, including “code ethnography” (Rosa, 2022), policy ethnography, ethnography of documentation, and ethnography of infrastructure (Star, 1999): open science, in other words, may be a great gift to ethnographers of science. By treating calls and requirements for open science, data-sharing, and transparency as not just methodological, but also empirical matters, we gain new questions for analysis, new field sites, and opportunities to revisit and reexamine core questions in the ethnographic study of science.

This article has suggested a wide array of such questions that ethnographers might pursue if we treat the emergence, embrace, and negotiation that surround calls for open science as a fruitful site of disruption from which to track contemporary contests over epistemic virtue. These questions are uniquely suited to ethnographic methods, focused as they are on not only what important actors in open science debates say—how scientists describe their own understandings of open science, or how funders’ characterize the merits of data-sharing or their own goals in promoting open science—but on what these actors do in the course of their day-to-day work. I have suggested, in other words, that we built on interview- or discourse analysis-based research about open science by observing processes, practices, and creative and intended uses of open science instruments and infrastructures, analyzing action by both those who understand themselves to be part of an open science movement and those who open science advocates hope might act on calls to recognize good knowledge on the basis of data-sharing.

Similarly, studying open science with an eye to its indeterminacy in this way can take ethnographers to a broad range of *field sites*. Certainly the sites these questions have suggested—those where researchers, journal editors, legislators and regulators gather to make and use knowledge—are ones where we might find rich action to observe. But in addition to philanthropists’ meetings, policy-makers’ gatherings, and researchers’ laboratories, conferences, and trainings, ethnographers of open science might visit other sites. If, following STS inquiries into the creative shaping of technologies by users (see, for instance, Oudshoorn and Pinch, 2003), ethnographers of open science might examine the ways that users actually interact with technologies from COS’s Open Science Badges and platforms like Dataverses and Open Science Framework to scoring and verification tools like SciScore or penelope.ai, we might also turn our attention to the software developers and engineers who build these platforms. The advent of open science also offers an opportunity for ethnographers of science to revisit one of its central questions: that of inscription. How have new epistemic virtues, data-sharing practices and open science mandates changed—or not changed—the ways laboratory scientists engage in “coding, marking, altering, correcting, reading, and writing” (Latour and Woolgar, 1979, 49), not to mention image-making (Daston and Galison, 2007; Vertesi, 2015)? Organizational ethnographers can treat institutions that interact with the open science movement—from IRBs to data repositories and libraries, think tanks like COS to state agencies like NIH or NSF—as field sites from which to observe the negotiation of a new set of normative understandings, platforms, tools, and policies, embracing the profusion of social and cultural action that occurs in these sites as worthy of our ethnographic curiosity. Studies like these will be chances for ethnographers to ask a broader range of questions about how the politics and practices of truth and facticity are shifting across a range of communities of knowers and social institutions in the wake of calls for open science.

If ethnography is good for the study of open science, studying open science is also good for ethnography. Studying open science can help ethnographers do three things: first, it can help us to think about the status of our own methods. Second, studying the process of open science can help inform ethnographers’ own decisions about disclosure of research materials. Third, the lines of inquiry opened by studying open science can point ethnographers toward a more general reinvigoration of studies of norms, values, and understandings of facticity in both politics and science.

Ethnographers considering calls to share research materials as demonstrations of transparency have often pointed out that ethnographic research by nature permits less of what Victoria Reyes calls “one-size-fits-all” approaches to disclosure. At times, as Reyes writes, “the very reason researchers and participants were able to talk about a certain subject was because of the promise of confidentiality” (Schultz, 2019, 188). Reyes suggests that ethnographic transparency might look less like release of primary data by default and might look more like transparency about the process of data analysis, as well as transparency about the decisions involved in whether and how particular people and places are identified and deidentified (Reyes, 2018). Yet as funders, publishers, and disciplinary associations increasingly specifically require data-sharing practices associated with open science as markers of transparency, ethnographers who decline to deploy those practices and interpret transparency otherwise may increasingly hazard disciplinary marginalization (Murphy et al., 2021, 42). Studying open science ethnographically can certainly help us understand these tradeoffs. But recalling how open science advocates’ definitions of legitimacy and epistemic virtue on the basis of particular practices of disclosure speak to both other researchers and to policy-makers, we can see how studying open science might help us to think about the political status of our work. If ethnographers decline, for instance, to share field notes, do we risk not only a loss of legitimacy among other scholars, but also reduced opportunities for our research to influence public decision-making? Better understandings of just how mandates and standards for data-sharing are being used in scientific and policy-making spaces can help us to consider these questions for our own work.

By observing the ways that conversations about open science are also conversations about how to recognize credibility or facticity, we can gain crucial perspective on the relationships between open science visions of knowledge and their visions of order. This perspective can also inform our methodological decision-making in essential ways, allowing us to make well-informed choices about how to set standards for transparency that remain faithful to the commitments to disrupt dynamics of exploitation and domination that emerged from ethnography’s first reckoning. If we are attuned to the relationship between the matter of who decides what makes for credible knowledge and the matter of who decides what makes for a good social policy, we can build ethnographic practices of revelation and concealment that build, rather than disrupt, the power—as both knowers and political actors—of the communities where our research occurs. If we want more democratic knowledge-making to emerge from ethnography’s reckoning with open science, we must consider these matters.

Looking to our own methods can also support ethnographers in generating principles of disclosure that enable accountability, empirical precision and contextualization, fruitful reanalysis, methodological training and innovation, and clarity of inference (Pool, 2017, Reyes, 2018, 208–13, 17)—and that are informed by the rich tradition of ethnographic studies of knowledge and proactively support the power of the communities where our research unfolds as knowers and actors. We can learn from examples of open science and data sharing projects in methodological and disciplinary locations like digital anthropology, environmental justice and environmental health research, STS, Indigenous studies, and decolonial science studies. These might include experiments like those of the Platform for Experimental Collaborative Ethnography and STS Infrastructures, an instance of PECE, that work to treat data as “entangled in systems of relations” and to “acknowledge the ways power is woven into language, common sense, and communicative practice” (Fortun et al., 2016, 18; Okune et al., 2022, 7). Inviting varied ways to contextualize data and building “interpretive annotation” into a recursive process of sharing data—and, through researcher reflection, making more—data, these platforms aim to encode ethnographic sensibilities in data-sharing infrastructures (Fortun et al., 2016, 16; Okune et al., 2022, 3–5).

They might also include practices of “strongly participatory” science: research processes that aim to enhance what Barbara Allen calls knowledge justice by bolstering laypeoples’ power to make claims and advance their visions for their own communities through their participation in scientific work (see Visvanathan, 2005; McCormick, 2009; Allen, 2018). We might also learn from the research methods and data governance models developed by the “civic science” Public Lab for Open Technology and Science, which formed in the wake of the BP oil spill using a model of local chapters. PLOTS has developed what co-founder Shannon Dosemagen and her coauthors call “situated” data-production and data-sharing practices, taking moments in which powerful actors such as police indicate interest in their work as cues that “recursive” refinement of these practices is necessary to reemphasize “the need of vulnerable communities to maintain control over representations of their territory” (Dosemagen et al., 2011). We can learn from these models, such as data trusts in which data are stewarded by a council whose role is defined by those data’s beneficiaries (Dosemagen and Tyson, 2020). Max Liboiron, founder of the Civic Laboratory for Environmental Action Research, writes of this as a question of “tak[ing] up science that enacts good Land relations” (Liboiron, 2021, 22). In CLEAR’s marine science and pollution research, this means that Indigenous groups not only make the invitations that begin any given research project and set the priorities and research questions to be investigated, but also own project data (Liboiron, 2016). Meanwhile, at the Environmental Justice Lab directed by STS scholar Michelle Murphy, the organization of data storage and translation in a pollution reporting application is determined according to frontline community useability (see Pollution Reporter, n.d.).

Ethnography’s reckoning with data collection and data-sharing, as Murphy, Jerolmack and Smith have written, both comes on the heels of and overlaps with another, “first reckoning”: the conversations about power, ethics, reflexivity, and representation that began in the 1980s and 1990s and continue today (Murphy, Jerolmack and Smith, 42–3). Through calls among ethnographers to democratize or horizontalize knowledge production by reversing power dynamics between researchers and non-academics, this earlier reckoning resulted in disciplinary standards requiring that ethnographers robustly consider the ways their own subjectivities and structural positions might influence the knowledge they produce. It also pushed ethnographers to question the notion that highly-trained outsiders should be treated as more authoritative knowers than members of communities themselves. In deciding how research practices are chosen, how data are arranged and stored, and who will “own” data or decide about its use, all of these models begin from epistemic virtues that are informed by ethnographic study of scientific practice and recall the insights of ethnography’s “first reckoning.” These models presume that the “the best research done in the best way,” to quote my open science workshop’s instructors, might not be characterized by the “default” assumption of “direct access” to all research materials for which the Center for Open Science’s mission statement calls. Instead, they situate the question of when and how to share data as one decision among many, all of which ought to be guided by awareness of the ways that claims to objectivity in science have been weaponized in service of maintaining violent arrangements, and by awareness of the impossibility of scrubbing the social from the scientific.

We also gain a different fundamental approach to facticity when we do not just try to achieve it, but treat it as an object of analysis. Contests over truth and credibility are, of course, a highly contentious and intensely politicized matter in our current moment, and there is a great deal of action for ethnographers to observe beyond the laboratory, the academy, or the scientific realm. Our methods are well-suited to investigating the cultural purchase, legal codification, and community negotiation of highly-politicized misinformation and disinformation about matters like abortion, electoral fraud, COVID-19, trans healthcare, or the post-2020 crime panic. Importantly, they are also well-suited to investigating the ways that policy-making and political debate about these matters raise questions for a wide array of actors about what makes for knowledge that is good enough to use in governance. Ethnographic studies can play an important role in giving us better analytic purchase on our current epistemic moment. If we treat controversies over the right way to know as moments of analytic opportunity and ask what is shifting about epistemic virtue and the ways that facticity is recognized, demonstrated, and contested, we gain opportunities to make ethnographic contributions of urgent importance.

## Author contributions

The author confirms being the sole contributor of this work and has approved it for publication.

## Footnotes

^1^While this article encourages ethnographers to undertake systematic studies of sites like this workshop, I did not myself do so. Because the descriptions I offer here are reflections on my experiences and the questions I later asked, not findings from systematic inquiry, my IRB determined that this project is not research involving human subjects and therefore did not need IRB review or approval (see VT IRB determination number 23–659; for more on exclusion from IRB review, see Lederman, 2004, 2007). However, I still checked in ahead of time with workshop organizer/trainers to let them know I was considering writing about my experiences in the workshop and to ask if there was anything they’d like me to keep in mind or to do or not do as I participated and wrote. On the day of the workshop, I included in my introduction to the group a note that I planned to write about the workshop, but not to identify anyone by name.

^2^Our instructors differentiated reproducibility from replicability: they explained that reproducible research is identified by the ability of another researcher to apply the same methods to the same materials, or to similar, pre-existing data, to achieve the same results. Replicable research, on the other hand is identified by the ability to implement the same methods for data collection and analysis to arrive at the same results. It is a matter, they told us, of the difference between internal and external validity: “You can reproduce something,” we learned, “that was wrong in the first place.”

^3^Together, these three sources account for $4,693,168, or over 85%, of the organization’s $5,468,200 budget—and the Templeton foundations and Arnolds for over 50% (COS Form, 2020). The Laura and John Arnold Foundation was the largest funder, contributing $2,289,280; DARPA accounted for $1,887,072; the John Templeton Foundation donated $170,561, the Templeton Religious Trust $163,352, and the Templeton World Charity Foundation $182,903. The charities Templeton founded prioritize projects concerning religion and science, including some that have been the subject of some controversy in fields like biology and religious studies (see, for instance, Wiebe, 2009; Waldrop, 2011), as well as projects advancing free markets, such as prominent right-wing think tanks like the Cato Institute and Mercatus Center (see Templeton Foundation and Templeton World Charities; Grant Database, n.d.). Laura and John Arnold, meanwhile, are Houston billionaires—John, profiled in the book on which the Oscar-winning documentary *The Smartest Guys in the Room* was based, is a former executive at Enron. After Enron’s collapse, he went on to found the hedge fund Centaurus Advisors (Taibbi, 2013). The Arnolds have joined Warren Buffet and Bill Gates in pledging to donate the majority of their wealth (Laura and John Arnold. The Giving Pledge, n.d.).
